# Cerebellar control of fear learning via the cerebellar nuclei–Multiple pathways, multiple mechanisms?

**DOI:** 10.3389/fnsys.2023.1176668

**Published:** 2023-05-09

**Authors:** Julie D. Urrutia Desmaison, Romain W. Sala, Ahsan Ayyaz, Pimpimon Nondhalee, Daniela Popa, Clément Léna

**Affiliations:** Neurophysiology of Brain Circuits Team, Institut de Biologie de l’Ecole Normale Supérieure (IBENS), Ecole Normale Supérieure, CNRS, INSERM, PSL Research University, Paris, France

**Keywords:** cerebellum, fear learning, prediction error, 4 Hz oscillations, ventrolateral periaqueductal grey, mediodorsal thalamus

## Abstract

Fear learning is mediated by a large network of brain structures and the understanding of their roles and interactions is constantly progressing. There is a multitude of anatomical and behavioral evidence on the interconnection of the cerebellar nuclei to other structures in the fear network. Regarding the cerebellar nuclei, we focus on the coupling of the cerebellar fastigial nucleus to the fear network and the relation of the cerebellar dentate nucleus to the ventral tegmental area. Many of the fear network structures that receive direct projections from the cerebellar nuclei are playing a role in fear expression or in fear learning and fear extinction learning. We propose that the cerebellum, via its projections to the limbic system, acts as a modulator of fear learning and extinction learning, using prediction-error signaling and regulation of fear related thalamo-cortical oscillations.

## 1. Introduction–Current understanding of fear learning and its neural representation

Fear is an unpleasant and often strong emotion caused by anticipation or awareness of danger in mammals, and its proper tuning is crucial for survival. As most emotions, fear is reflected in hormonal, behavioral and physiological responses. It can be innate or learnt (LeDoux, 2012). Memories and perception of fear are flexible and entrain behavioral adaptive changes based on previous experience (Davis, 1992). As fear is a powerful sensation, it has a strong influence on the psychological wellbeing in humans. Dysfunction in fear processing in humans leads to emotional disorders widely spread in the population such as anxiety disorders, post-traumatic stress disorder or depression. Understanding the neurophysiological basis of how fear memories are formed and modified is an important step toward targeted therapies of these disorders.

Recent studies have provided us with a model of the brain structures that are involved in fear learning and extinction (Tovote et al., 2015). This model is based on associative fear learning in pavlovian fear conditioning (Pavlov, 1927). It proposes that fear is mediated by the amygdala and that the hippocampus and medial prefrontal cortex are involved in its consolidation. In addition, midbrain structures, most notably the hypothalamus and the periaqueductal gray, are involved in generating autonomous, active and passive fear reactions (Signoret-Genest et al., 2023).

The influence of cerebellar lesions on fear related behavior was first described in rodents, felines and primates (Reis et al., 1973; Berman et al., 1974; Supple et al., 1987). Since the description of the cerebellar cognitive affective syndrome by Schmahmann and Sherman (1997), we know that cerebellar lesions lead to altered emotional behavior in humans. While this cerebellar involvement in affective activity has been established in patients more than 20 years ago and has received a strong support from many fMRI studies (Stoodley, 2012) and is supported by animal experiments (Sacchetti et al., 2002; Strata et al., 2011), the nature and mechanisms of this involvement remain largely elusive.

The cerebellum is anatomically connected through one or two synapses to many important structures in fear learning, e.g., it sends monosynaptic projections to the ventrolateral periaqueductal gray (Frontera et al., 2020) and to thalamic areas projecting to the basolateral amygdala (Jung et al., 2022) and prefrontal cortex (Groenewegen, 1988; Hintzen et al., 2018; Frontera et al., 2023), while the cerebellar cortex receives emotion-related input via the basilar pontine nucleus (Farley et al., 2016) and the inferior olive (Watson et al., 2009; Kostadinov et al., 2019). Still, evidence on the exact influence of the cerebellum has only emerged recently.

The cerebellum is likely to be involved in both fear learning and fear response, as it also influences autonomous regulation (Chen et al., 1994) and motor control (Ghez and Fahn, 1985). In associative fear learning (as in pavlovian fear conditioning), a fear response toward a before neutral stimulus is elicited only after the association of the neutral stimulus to a fearful event (Pavlov, 1927). Hence, in this setting, fear learning is causative of the fear response. In this review, we will discuss the role of the cerebellum, in particular the cerebellar nuclei, during the process of fear learning and extinction learning. We will:

(1) Introduce some main concepts related to associative fear learning.
(2) Discuss the anatomical connections of the cerebellar nuclei to associative fear learning related structures and their impact on behavior.
(3) Theorize potential mechanisms of information transfer from the cerebellar nuclei to the limbic system.
(4) Hypothesize about the content of the information sent to the limbic system from the cerebellar nuclei during associative fear learning.

### 1.1. Investigating fear learning–Simple associative learning as a model for fear acquisition and extinction

Pavlovian fear conditioning is the most commonly used experimental paradigm for fear learning and extinction, which has provided the main support for current concepts about the neural processes underlying fear learning, although it might be better referred to as “threat conditioning” (LeDoux, 2014).

Pavlovian fear conditioning (Pavlov, 1927) allows us to access fear learning as a form of simple associative learning. This paradigm is widely used in the literature as a straightforward experimental approach for studying the neuronal substrates of emotional associative learning and the mechanisms of aversive memory formation (e.g., Rescorla and Wagner, 1972; Johansen et al., 2010; Dejean et al., 2016; Wright and McDannald, 2019; Frontera et al., 2020).

In Pavlovian fear conditioning, a neutral conditioned stimulus acquires the ability to elicit fear responses after pairing with a aversive unconditioned stimulus (usually a foot shock). After this pairing, repeated unpaired presentations of the conditioned stimulus gradually yield a decrease of the fear responses in a process known as “extinction learning,” that results from the formation of a new memory competing with the original conditioned stimulus-unconditioned stimulus association (Pavlov, 1927; Myers and Davis, 2002).

Fear learning and fear extinction learning are caused by the association between conditioned stimulus and unconditioned stimulus, or by the repeated presentation of the conditioned stimulus alone without the previously associated unconditioned stimulus, respectively. Due to the association process in fear conditioning, the formerly neutral conditioned stimulus gains a predictive value: it becomes a fearful stimulus, as it has become a predictive signal for the occurrence of the aversive unconditioned stimulus. Another layer of learning is added in fear extinction, where the conditioned stimulus is consequently presented without the aversive stimulus. This leads to association of the conditioned stimulus to safety.

In order to access the fear experienced by the animal, one may rely on the animal’s visible behavior or on physiological reactions. Most rodents respond to aversive situations with a certain pattern of fear behavior. In mice, freezing, startling, grooming, vocalization, littering and urinating are common fear reactions (Blanchard and Blanchard, 1988; Blanchard et al., 1995). Freezing behavior in particular is a very stable behavior in mice confronted to an unavoidable aversive stimulus, such as a foot shock or the presentation of predator odor.

Freezing has largely been used as an indicator of fear in rodents over the last decades (Johansen et al., 2010; Dejean et al., 2016; Frontera et al., 2020; Lawrenson et al., 2022). Still, measuring freezing alone bears some disadvantages: firstly, it is difficult to differentiate freezing from simple immobility. Secondly, the absence of freezing is not a proof for the absence of fear, as fear might also be expressed by a flight reaction (Lang et al., 2000). To have a more precise evaluation of rodent fear, freezing measures can be combined with the measure of physiological parameters such as the heart rate (Tovote et al., 2005; Signoret-Genest et al., 2023) or the occurrence of a startling response (VanElzakker et al., 2014).

### 1.2. Prediction error induces stimuli association in both reward and aversive conditioning

Associative learning takes place when a positive (e.g., reward) or negative (e.g., aversive) reinforcer occurs in relation with a given sensory configuration. How does the brain process this information ? Rescorla and Wagner have proposed in 1972 that the rate of learning is determined by the unexpectedness of the association of sensory stimuli with the presence (or omission) of a negative or positive reinforcer. In their reasoning, this unexpectedness, termed as prediction error, is acting as a learning signal driving associative learning (Rescorla and Wagner, 1972).

There are different types of prediction errors, as its principle applies for different outcome modalities. If a sensory output is different than expected, this would be referred to as a sensory prediction error (review: Wolpert et al., 2011). One of the most studied examples of sensory prediction error is eye-blink conditioning. Here, the -aversive- sensory prediction error is evoked by an unexpected mildly aversive stimulus (air-puff); if predictive sensory cues are available and elicits a conditioned sensory-motor response (eye-blink) to the sensory cues (Cason, 1922). A neuronal coding of such sensory prediction error has been found to be encoded by climbing fibers in the cerebellum and to drive sensorimotor learning (Wolpert et al., 1998; Tseng et al., 2007). Different from the sensory prediction error, an emotional prediction error occurs if the emotion associated with the outcome differs from the expected emotional outcome. This type of prediction error occurs for both rewarding and aversive stimuli. It was described in the limbic system (Barto, 1995; Montague et al., 1996) and leads to emotional learning, provided cues are available paired to the rewarding/aversive stimuli. In the following, we focus on emotional prediction errors in the limbic system (either aversive or reward prediction error) as we discuss fear and extinction learning.

Rescorla and Wagner’s theory applied to fear conditioning predicts that an unexpected unconditioned stimulus following a conditioned stimulus, or the omission of an expected unconditioned stimulus previously associated with the conditioned stimulus, would generate a prediction error. The impact of this prediction error on the learning rate is determined by its size. The size of the prediction error is based on the difference between the actual unconditioned stimulus and the expected unconditioned stimulus based on the memory associated with the sensory stimuli from former trials. Thus, prediction error size changes over the course of repetitive conditioned stimulus-unconditioned stimulus parings, as with each pairing the learning progresses and the conditioned stimulus conveys a stronger predictive signal of the unconditioned stimulus. As a result, the learning grows smaller with each pairing trial until the association is complete. In addition, the prediction error is influenced by a set of variables specific to each experimental setting.

In the paradigm above, accessing the neural representation of the prediction error shall provide access to the neural basis of fear learning. Prediction error signals have been observed in both reinforced learning and fear conditioning, in multiple structures linked to the limbic system. For instance, prediction error was described during reward conditioning in the central amygdala (Lee et al., 2010) and during fear conditioning in the lateral amygdala and also in the ventrolateral periaquecductal grey (Johansen et al., 2010). Prediction error is likely to be generated by dopaminergic neurons in the midbrain during conditioning (Barto, 1995; Montague et al., 1996) as they have been found to be responsive upon the presentation of unexpected stimuli.

Different concepts are associated with dopaminergic neuron firing behavior: they either accelerate or decelerate their firing rates on unexpected reward presentation or omission (“Signed prediction error,” Klavir et al., 2013; Keiflin et al., 2019), or they simply accelerate firing once an unexpected event happens (“Unsigned prediction error,” Pearce and Hall, 1980; Takeuchi et al., 2016; Salinas-Hernández et al., 2018). Dopaminergic neurons can be found in different structures related to fear and reward learning and receive multiple input from extra-limbic structures. It has been confirmed that during fear learning, prediction error signaling is present in different structures within the fear network that are potentially communicating and updating each other (review: McNally et al., 2011). The cerebellum is one of the structures that sends input to dopaminergic neurons which convey prediction error (Carta et al., 2019; Frontera et al., 2020; Vaaga et al., 2020) and could thus participate to their computation.

### 1.3. Brain oscillations are associated with fear expression and conditioning

Information processing in the brain relies on electrical activity. Early electrophysiological studies in animals and humans revealed the presence of oscillatory patterns of neuronal activity in the brain (Beck, 1890; Berger, 1930). These oscillatory patterns have been shown to arise from the synchronization of large populations of neurons (Singer, 1999), and have been theorized to be critical for information transfer between brain regions (Averbeck and Lee, 2004; Fries, 2005). Moreover, they have been shown to support functions such as memory consolidation (Diekelmann and Born, 2010; Penagos et al., 2017).

More recently, different cognitive and emotional processes have been associated with olfactory-driven oscillations (Heck et al., 2019; Folschweiller and Sauer, 2021). While they were originally detected in olfactory-related structures such as the olfactory bulb and the pyriform cortex (Freeman, 1960; Boeijinga and Lopes da Silva, 1988), they have been later observed in the hippocampus, while the oscillatory coherence between the olfactory bulb and the hippocampus was correlated to the performances of mice in an odor memory discrimination task (Kay, 2005). Strikingly, low frequency oscillations phase-locked to breathing are present in the dorso-medial prefrontal cortex during the expression of the freezing fear behavior following fear conditioning in mice (Dejean et al., 2016; Karalis et al., 2016; Bagur et al., 2021). These olfactory-driven oscillations have a dominant frequency around 4 Hz (Karalis et al., 2016; Bagur et al., 2021), and were shown to be sufficient to induce the expression of freezing.

Overall, the findings summarized above show that emotional learning is tightly associated with the concept of prediction error, which is encoded by dopaminergic neuron activity, while strong evidence links brain oscillations and emotional processing.

### 1.4. Basic concepts of cerebellar functioning

The cerebellar cortex has a particular anatomical architecture that allows rapid parallel computation of multiple processes. It is a three-layer cortex functioning roughly as a two-layer feed-forward network where granule cells in the granule layer receive precerebellar inputs via mossy fibers, perform re-encoding of the incoming information and project via parallel fibers to Purkinje cells. Associative learning takes place in Purkinje cells, notably driven by climbing fiber inputs from the inferior olive. Purkinje cells form the sole output of the cerebellar cortex and project to the cerebellar nuclei, which in turn project to downstream structures. The cerebellar cortex is functionally organized in multiple zones (Andersson and Oscarsson, 1978; Apps and Garwicz, 2005; Apps and Hawkes, 2009; Voogd, 2011), each containing multiple microzones (Oscarsson, 1979) which are connected to distinct areas in the cerebellar nuclei who in turn are projecting to distinct areas of the brain (Diedrichsen et al., 2011; Watson et al., 2014).

The cerebellar nuclei are integrating the output of the cerebellar cortex with information from other brain areas to which they are also projecting (Eccles et al., 1967; Marr, 1969; Albus, 1975). This allows the cerebellum to form a loop with other brain areas and to shape their output via recurrent loops (reviews: Person and Khodakhah, 2016; Tanaka et al., 2020). Cerebellar function has been extensively studied in motor function and learning but less in emotional learning, despite the existence of multiple anatomical connections between the cerebellum and structures in the limbic system and the known impact of cerebellar lesions on emotional behavior (Groenewegen, 1988; Schmahmann and Sherman, 1997; Sacchetti et al., 2002; Watson et al., 2009; Strata et al., 2011; Stoodley, 2012; Farley et al., 2016; Hintzen et al., 2018; Kostadinov et al., 2019; Frontera et al., 2020, 2023; Jung et al., 2022).

In light of several recent findings, we shall discuss in the present review how the cerebellum is intertwined with different types of transfer of emotional information to the limbic system and may participate to emotional learning.

## 2. Anatomical and behavioral evidence–The cerebellar nuclei in the fear network and fear learning

**FIGURE 1:**
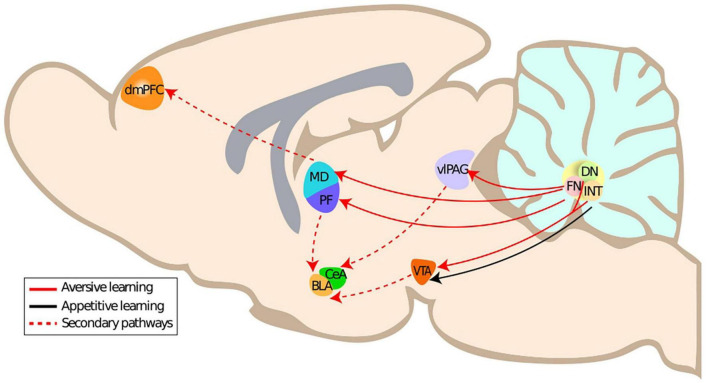
Primary and secondary anatomical connections of the cerebellar nuclei to aversive learning related structures. DmPFC, dorsomedial prefrontal cortex; CeA, central amygdala; BLA, basolateral amygdala; PF, parafascicular thalamus; MD, mediodorsal thalamus; FN, fastigial nucleus; vlPAG, ventrolateral periaqueductal gray; ITN, interposite nucleus; DN, dentate nucleus.

The neuronal structures responsible for fear learning and expression constitute a dynamic network that is continuously monitoring the environment in order to adjust behavior (Tovote et al., 2015). This network includes the amygdala (central nucleus of the amygdala), hippocampus, medial prefrontal cortex, the periaqueductal gray, and most notably the ventrolateral periaqueductal grey in the case of freezing (Tovote et al., 2015; McNaughton and Corr, 2018), the cerebellar vermis and one of the cerebellar nuclei, the fastigial nucleus (Strata et al., 2011; Koutsikou et al., 2014; Frontera et al., 2020; Lawrenson et al., 2022). In this section, we will present the evidence on the anatomical connections of the cerebellar nuclei, in particular the fastigial nucleus, to the fear network and their impact on fear learning and fear extinction. We will focus on the impact of the multiple projections originating from the fastigial nucleus to the fear network (Figure 1), which are thus notably relevant for aversive learning.

### 2.1. The fastigial nucleus is connected to the fear network via multiple pathways

The fastigial nucleus is part of the cerebellar nuclei [in rodents: fastigial nucleus, interposed nucleus, and dentate nucleus] and relays cerebellar cortex outputs. It has been associated with both motor function, e.g., eye movement but also with autonomous and emotional functions. The fastigial nucleus is receiving afferents not only from the cerebellar cortex, the vestibular nuclei and inferior olive but also from other structures such as the hypothalamus and raphe nuclei (Yu and Wang, 2022). The fastigial nucleus sends efferents to the ventral periaqueductal gray, the thalamus (e.g., Mediodorsal thalamus, parafascicular nucleus) (Batton et al., 1977; Frontera et al., 2020; Vaaga et al., 2020) and the superior colliculus (Fujita et al., 2020). The existence of distinct neuronal populations with specific connectivity supports the existence of multiple functional modules in the fastigial nucleus (Fujita et al., 2020).

While projections from the cerebellar nuclei to limbic areas of the brain have been described in the 1970‘s (Harper and Heath, 1973; Heath and Harper, 1974) and the impact of lesions in the cerebellar vermis on affective functions (Schmahmann syndrome) was established in the 90’s (Schmahmann and Sherman, 1998), it was not until recently that modern techniques, such as the introduction of targeted chemo- and optogenetic strategies, have made it possible to investigate the impact of the fastigial nucleus projections on fear learning and fear extinction.

### 2.2. Fastigial nucleus to ventrolateral periaqueductal gray projections—A pathway influencing fear learning

#### 2.2.1. Fastigial nucleus-ventrolateral periaqueductal gray projections are altering fear memories

The caudal fastigial nucleus is sending a large amount of projections to the ventrolateral periaqueductal grey. These neurons are mostly contacting dopaminergic neurons (Vaaga et al., 2020) and glutamatergic neurons, but also on GABAergic neurons within ventrolateral periaqueductal grey (Frontera et al., 2020).

In rodents, the manipulation of fastigial nucleus - ventrolateral periaqueductal grey neurons projecting to the ventrolateral periaqueductal grey leads to modified fear memories and learning. One study used viral expression of DREADDS in fastigial nucleus - ventrolateral periaqueductal grey-projecting neurons in mice to manipulate activity at defined time points of a fear conditioning and extinction paradigm. Excitatory DREADD activation during fear conditioning led to decreased fear memories the following days and thus faster extinction, while inhibitory DREADDS activation led to increased fear memories (and slower extinction). Optogenetic stimulation of the fastigial nucleus - ventrolateral periaqueductal grey pathway during fear conditioning solely at the time of the unconditioned stimulus (where prediction error shall take place) led to decreased fear expression during extinction (Frontera et al., 2020).

In another study, inhibitory DREADD activation during fear conditioning in rats led to decreased fear extinction learning rates (Lawrenson et al., 2022) consistent with the results of Frontera et al. (2020). Interestingly, fastigial nucleus inactivation using GABA_*A*_-agonist muscimol during fear consolidation led to increased freezing period duration in extinction learning suggesting also an offline contribution of fastigial nucleus activity (Lawrenson et al., 2022).

What could be the neural mechanism behind this influence on fear learning and fear extinction learning? To address this question, we highlight some functions of the ventrolateral periaqueductal grey in the fear network in the following section.

#### 2.2.2. The ventrolateral periaqueductal gray participates in fear learning with prediction error signals

The periaqueductal gray is located in the midbrain around the aqueduct from the third to the fourth ventricle. It is organized in four columns each playing a distinct role in behavior or signal processing, and having distinct afferent and efferent connections (McNally et al., 2011). The ventrolateral periaqueductal grey is particularly involved in passive threat response (Carrive, 1993; Koutsikou et al., 2014); it controls motor behaviors, such as immobility or freezing, and several other autonomic responses, via projections to the brainstem.

Ventrolateral periaqueductal gray has long been considered a structure only responsible for passive fear responses (Liebman et al., 1970; Bandler et al., 1985; Carrive, 1993; Fanselow, 1994). Within the last decade, it has been identified as a pivotal structure in fear and fear extinction learning (Ozawa et al., 2017; Groessl et al., 2018; Frontera et al., 2020; Vaaga et al., 2020).

Ventrolateral periaqueductal gray has been reported to generate, in the context of fear conditioning, a predictive signal in response to a conditioned stimulus that indicates an upcoming aversive event. This predictive signal is represented by an increase of firing activity after the conditioned stimulus-onset that increases over repetitions of conditioned stimulus-unconditioned stimulus pairings, and which reflects conditioned stimulus-unconditioned stimulus association (Johansen et al., 2010). There is evidence that ventrolateral periaqueductal grey also encodes prediction error. A reciprocal pathway connects ventrolateral periaqueductal grey and the central nucleus of the amygdala in which dopaminergic neurons from the ventrolateral periaqueductal grey seem to encode prediction error during fear conditioning (Groessl et al., 2018). In agreement with the prediction error theory, signals from dopaminergic neurons in ventrolateral periaqueductal grey responding to the unconditioned stimulus decrease with each conditioned stimulus-unconditioned stimulus pairing in fear conditioning. Concordant to these findings, glutamatergic neurons in ventrolateral periaqueductal grey have been shown to produce predictive feedback in fear learning. The inhibitory input from the central nucleus of the amygdala to the ventrolateral periaqueductal grey would thus be modulated in function of the unexpectedness of a unconditioned stimulus (Ozawa et al., 2017; Massi et al., 2023). This supports the hypothesis of prediction error computation in ventrolateral periaqueductal grey fear learning.

In light of the bidirectional influence of the fastigial nucleus - ventrolateral periaqueductal grey projections on fear learning, we may thus hypothesize that the fastigial nucleus contributes to the modification of the prediction error signaling in the ventrolateral periaqueductal grey with an increase of fastigial nucleus inputs indicating a stronger expectation of the unconditioned stimulus (hence weaker learning following unconditioned stimulus actual occurrence) and a decrease of fastigial nucleus inputs reducing the expectation (and thus reinforcing the learning). This hypothesis remains, however, to be investigated in targeted experiments in the fastigial nucleus and ventrolateral periaqueductal grey.

### 2.3. The fastigial nucleus to mediodorsal thalamus pathway influences fear extinction learning

While the fastigial nucleus of the cerebellum is known to project to motor nuclei of the thalamus, it has also been shown to send projections to other non-motor thalamic nuclei, such as the mediodorsal thalamus (Groenewegen, 1988; Fujita et al., 2020). Indeed, Groenewegen has shown that the injection of horseradish peroxidase in the mediodorsal thalamus thalamus of the rat would induce a profuse retrograde labeling in the contralateral fastigial nucleus (Groenewegen, 1988). This observation was later supported by Fujita et al. (2020) who observed robust projections of the fastigial nucleus to the mediodorsal thalamus thalamus using an anterograde viral approach.

The mediodorsal thalamus is highly embedded in the limbic network, and is notably connected to the dorso-medial prefrontal cortex (Hunnicutt et al., 2014; Alcaraz et al., 2016) and the baso-lateral amygdala (Hintiryan et al., 2021). Consistent with an involvement of both dorso-medial prefrontal cortex and baso-lateral amygdala in fear extinction (Herry and Garcia, 2002; Vidal-Gonzalez et al., 2006; Giustino and Maren, 2015; Tovote et al., 2015), the mediodorsal thalamus has been shown to also contribute to fear extinction (Lee and Shin, 2016).

The mediodorsal thalamus is able to drive plasticity in the dorso-medial prefrontal cortex (Herry et al., 1999), which is crucial for fear extinction (Herry and Garcia, 2002). Furthermore, neuronal activity in the mediodorsal plays a causal role on fear extinction, as Lee et al. (2012) were able to bi-directionally modulate fear extinction by modifying pharmacologically or electrically the firing patterns in the mediodorsal thalamus. While the induction of tonic firing in the mediodorsal thalamus during conditioned stimulus facilitated fear extinction, the induction of burst firing led to an impairment in fear extinction. Moreover, Paydar et al. (2014) observed that inhibiting MD by locally injecting an agonist of GABA_A_ receptors reduced fear extinction.

Thus, the fastigial nucleus stands out as a potential actor in the regulation of fear extinction through its projections to the mediodorsal thalamus. We recently showed in our laboratory, that chemogenetically inhibiting the projections from the fastigial nucleus to the mediodorsal thalamus during fear extinction leads to an impaired extinction learning (Frontera et al., 2023). This increased expression of fear behavior elicited by the presentation of the conditioned stimulus was associated to an increased bursting activity in the mediodorsal thalamus, consistent with the role of this firing pattern in preventing extinction learning (Lee et al., 2012). This strongly suggests that the cerebellum is crucial for the execution of fear extinction, and that the fastigial nucleus-mediodorsal pathway contributes to the regulation of this function.

### 2.4. Fastigial nucleus to superior colliculus projections in other forms of fear

#### 2.4.1. The fastigial nucleus projects to the superior colliculus

The superior colliculus is an area located in the midbrain of mammals, that consists of multiple layers. Many studies provide information about projections from the cerebellum to the superior colliculus. For example, Roldán and Reinoso-Suárez (1981), found cerebellar projections to the superior colliculus in the cat and described that the cerebellar nuclei project to the deep layer of superior colliculus, which is related to head orientation and eye movements. Kawamura et al. (1982), showed that the projections arising from the cerebellar nuclei originated from the caudal half of the fastigial nucleus and the ventrolateral part of the posterior interposed nucleus. Fujita et al. (2020), specified that the fastigial nucleus neurons in the caudal portion of a dorsolateral protuberance project to the superior colliculus and periaqueductal grey.

#### 2.4.2. The superior colliculus is involved in pathological fear consolidation and innate fear

The superficial layer of the superior colliculus receives input from the retina, that is exclusively related to visual stimuli. The deeper layers are more related to motor functions, especially eye movements. Visual stimuli are relevant for fear learning, which makes the superior colliculus a relevant structure for the association of a neutral stimulus to a fearful stimulus. The superior colliculus is also known to play a role in fear expression. More precisely, superior colliculus is part of the expression of visually transferred innate fear (Wei et al., 2015) and unconscious fear (Morris et al., 1999). Moreover, dopamine D2 receptors in both the dorsal periaqueductal gray and the superior colliculus seem to be controlling this innate fear (Muthuraju et al., 2016).

Therefore, the fastigial nucleus-superior colliculus pathway might be involved in the expression of fear, especially as the same cell populations in fastigial nucleus that are targeting the ventrolateral periaqueductal grey are also targeting the superior colliculus (Fujita et al., 2020). Further experiments are required to explore this possibility.

### 2.5. Cerebellar nuclei to VTA projections are implicated in both rewarding and aversive emotional learning

#### 2.5.1. Deep cerebellar nuclei to VTA monosynaptic projections influence emotions bidirectionally

Anatomical evidence shows cerebellar nuclei to ventral tegmental area mono-synaptic projections, which could be involved in emotional learning (Carta et al., 2019; An et al., 2021; Baek et al., 2022; Yoshida et al., 2022). Indeed, optogenetic stimulation of cerebellar nuclei to ventral tegmental area projections triggered place preference for compartments where stimulation occurred, indicating that the activation of this pathway is rewarding (Carta et al., 2019).

Another piece of evidence for the involvement of the cerebellar nuclei into reward processing in ventral tegmental area was presented recently by Baek et al. (2022). Investigating on depression-like behavior in mice, they proved that cerebellar nuclei to ventral tegmental area projections are also connected to aversive emotions. Starting from the same cerebellar nuclei-ventral tegmental area projections, they described a pathway originating from the cerebellar Crus I projecting to the dentate nucleus, which projects to the ventral tegmental area. Chemogenetic inhibition of Crus I—dentate nucleus projections in mice enduring a chronic restraint stress paradigm (repeated forced swimming tests and tail suspension tests) led to decreased immobility in both forced swimming tests and tail suspension tests. Even more striking, the chemogenetic stimulation of direct dentate nucleus to ventral tegmental area projections was sufficient to increase immobility in tail suspension tests and forced swimming tests (Baek et al., 2022). These results are very intriguing for two reasons: firstly, they imply that the ventral tegmental area is also influencing behavior linked to aversive emotions. Secondly, they indicate that this effect of ventral tegmental area is regulated by the cerebellum.

Overall, the cerebellar nuclei are interacting with the emotional learning system via both reward and aversion related structures. Still, the mere amount of cerebello-limbic interactions does not reveal the specific content of the information transferred from the cerebellar nuclei to the limbic system. To hypothesize about this informative content, it is necessary to investigate on the neural mechanisms coupling the cerebellar nuclei and the limbic system.

## 3. How is the cerebellum transferring information to the limbic system–A multiple mechanism approach?

To form hypotheses on how the cerebellum exerts influence over circuits involved in fear learning and extinction learning, we focus on two principal concepts of information processing relevant to emotion: prediction error and brain oscillations.

### 3.1. Cerebellar nuclei output in emotional prediction error and emotional expectation signaling

Ample evidence points toward a role of cerebellar computations of prediction error to achieve motor control (Popa and Ebner, 2019). In eye-blink conditioning, an aversive associative learning relying on cerebellar plasticity, the climbing fiber may not only encode aversive sensory prediction error but also a temporal difference prediction error (close to what is encoded in midbrain dopaminergic neurons) (Ohmae and Medina, 2015). Human imaging studies of the cerebellum in fear learning paradigm evidenced strong activation in aversive stimuli prediction after associative fear learning (Fischer et al., 2000), but more strikingly showed strong activations interpreted as fear prediction error signaling in response to unconditioned stimulus omission (Ernst et al., 2019). Since ventrolateral periaqueductal grey and ventral tegmental area both receive monosynaptic inputs from the cerebellar nuclei and are known for emotional prediction error signaling during fear and fear extinction learning, it is very likely that the cerebellum contributes to prediction error computations; this may notably take place in the ventrolateral periaqueductal grey where the fastigial nucleus - ventrolateral periaqueductal grey pathway exerts a bi-directional influence over fear learning (Frontera et al., 2023).

### 3.2. Information transfer via oscillatory activity–Oscillations might coordinate cerebellar structures with the limbic system during fear learning

The freezing fear response in rodents is closely related to limbic slow (∼4 Hz) oscillations. Indeed, Karalis et al. (2016) observed a strong temporal relationship between these oscillations and the expression of fear, as a rise of 4 Hz power in the local field potential of the dorso-medial prefrontal cortex was predictive of the onset of a freezing episode, and a decrease of 4 Hz power preceded the offset of a freezing episode. Furthermore, the generation of such oscillations using analog optogenetic stimulation was sufficient to elicit freezing (Karalis et al., 2016). A total of 4 Hz oscillations are able to organize the local neuronal activity in the dorso-medial prefrontal cortex (Dejean et al., 2016; Karalis et al., 2016), known to drive the expression of fear behavior (Courtin et al., 2014). These oscillations propagate to the basolateral amygdala, where they also organize local neuronal activity (Karalis et al., 2016; Ozawa et al., 2020). Ozawa et al. (2020) observed that the induction of 4 Hz oscillations in the baso-lateral amygdala was able to elicit the retrieval of a fear memory and to control the temporal distribution of freezing episodes, further supporting the role of these 4 Hz oscillations during fear extinction.

**FIGURE 2:**
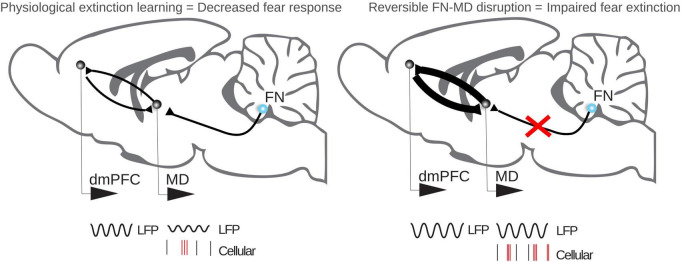
Fastigial nucleus—MD 4 Hz oscillations are impacting fear learning. In physiological fear extinction, FN regulates oscillatory activity in the MD, which in turn regulates oscillatory activity in the dorso-medial prefrontal cortex (dmPFC). Upon disruption of the FN-MD pathway, the oscillatory pattern in MD changes and fear extinction is impaired.

Until recently, little was known on the presence of these 4 Hz oscillations in other nodes of the limbic circuit. In a recent study (Frontera et al., 2023; Figure 2), we demonstrated the presence of 4 Hz oscillations in the mediodorsal thalamus during fear extinction, entrained by the dorso-medial prefrontal cortex. We also observed that dorso-medial prefrontal cortex 4 Hz oscillations modulate the occurrence of mediodorsal thalamus bursting, known to prevent fear extinction, suggesting an influence of dorso-medial prefrontal cortex 4 Hz oscillations on fear extinction. Moreover, we observed that the inhibition of fastigial nucleus - mediodorsal thalamus pathway during extinction leads to an increase in dorso-medial prefrontal cortex 4 Hz oscillations, an increased coherence between the dorso-medial prefrontal cortex and the mediodorsal thalamus in this range (2–6 Hz), and an improved phase locking of mediodorsal thalamus bursting to dorso-medial prefrontal cortex 4 Hz oscillations. This suggests that the cerebellum acts as a dampener of cortico-thalamic coupling of 4 Hz oscillations during the retrieval of fear memory, unstabilizing fear expression and facilitating fear extinction. In light of the role of midbrain dopaminergic neurons in fear extinction (Salinas-Hernández et al., 2018) and the presence of cerebellar input to this area (Carta et al., 2019), an intriguing possibility is that the cerebellum could also affect fear extinction via this pathway.

Beyond the scope of fear learning and expression, there is strong evidence on the cerebellar involvement in the coordination of 4 Hz oscillatory activity in multiple neural circuits. Evidence points toward Crus I and Lobulus simplex as important structures for cerebellum-driven coordination of oscillatory activity (Liu et al., 2022, review: McAfee et al., 2021). In cerebello-hippocampal interaction, Purkinje cell activity in both Crus I and Lobulus Simplex shows coupling to the phase of neural activity in the medial prefrontal cortex and cornu ammonis 1 in the range of delta oscillations (0.5–4 Hz) and for Lobulus simplex also in the range of gamma oscillations (25–100 Hz) (McAfee et al., 2019). The optical stimulation of lobulus simplex even impairs spatial working memory performance and medial prefrontal cortex—dorsal hippocampus oscillatory coherence in the gamma band (Liu et al., 2022). Also, Crus I and Crus II seem to influence the coherence of oscillatory activity between sensory and motor cortex in the theta and the gamma band, which points out that cerebellar coordination of oscillatory activity is a mechanism applied in multiple functions of the central nervous system (Popa et al., 2013; Lindeman et al., 2021).

## 4. Cerebellar nuclei-midbrain projections are likely to convey an emotional update to the limbic system

**FIGURE 3:**
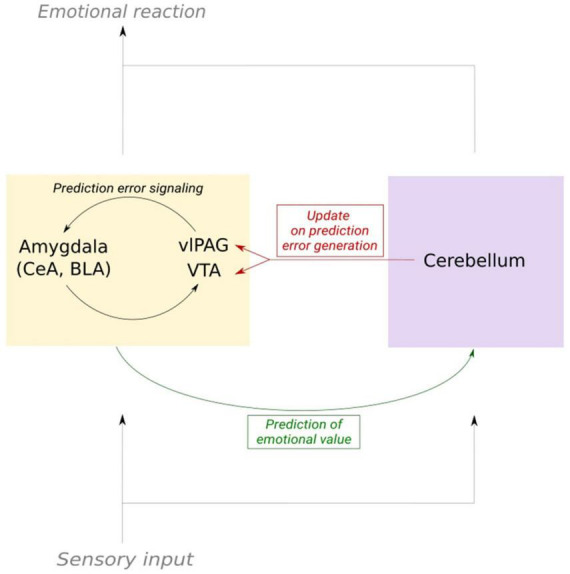
Schematic of the cerebellum as an updater of prediction error signaling in fear learning. Emotional prediction error is mediated by the limbic system, mainly by substructures in the amygdala, VTA and vlPAG. Copies of the sensory input that elicit an emotional response are sent to both the cerebellum and dopaminergic structures. This enables the cerebellum to send an updating signal to the dopaminergic system. In consequence, prediction error signaling and the prediction of the emotional value of a stimulus can be adapted.

In conclusion, this brief survey of the literature supports the hypothesis that the information on emotional value computed by the cerebellar cortex is sent to prediction error generating structures via the different cerebello-limbic pathways. Looking at the particular structure of the cerebellum and its role in motor learning, we hypothesize that the information delivered from the cerebellar nuclei to dopaminergic midbrain areas is acting as an updating signal on the emotional state (Figure 3). This theory needs experimental confirmation. We discussed multiple pathways relating the cerebellar nuclei to the limbic system. The multiplicity of the pathways coupling the cerebellum to the limbic system also suggests that multiple types of mechanisms contribute, as exemplified by the cerebellar modulation of fear extinction via the control exerted on thalamocortical oscillations. Decrypting the content of the cerebellar signal sent to higher brain regions during fear- and fear extinction learning is the next step toward confirming the theory of an emotional cerebellar updating signal.

## Author contributions

All authors contributed to the design and conceptualization, literature search, and writing of the review and approved the final submitted version.
